# Plant polyphenols as electron donors for erythrocyte plasma membrane redox system: validation through *in silico *approach

**DOI:** 10.1186/2191-2858-2-12

**Published:** 2012-04-04

**Authors:** Rajesh Kumar Kesharwani, Durg Vijay Singh, Krishna Misra, Syed Ibrahim Rizvi

**Affiliations:** 1 Division of Applied Science & Indo-Russian Center For Biotechnology [IRCB], Indian Institute of Information Technology, Allahabad 211012, India; 2 Department of Bioinformatics, UIET, CSJM University, Kanpur 208024, India; 3 Department of Biochemistry, University of Allahabad, Allahabad 211002, India

**Keywords:** *In Silico*, QSAR, Polyphenols, Pharmacophoric, Docking simulation, Glide, Molegro Virtual Docker

## Abstract

**Background:**

The plasma membrane redox system (PMRS) has extensively been studied in erythrocytes. The PMRS plays an important role in maintaining plasma redox balance and provides a protective mechanism against oxidative stress. Earlier it was proposed that only NADH or NADPH provided reducing equivalents to PMRS; however, now it is acknowledged that some polyphenols also have the ability to donate reducing equivalents to PMRS.

**Methods:**

Two different docking simulation softwares, Molegro Virtual Docker and Glide were used to study the interaction of certain plant polyphenols viz. quercetin, epigallocatechin gallate, catechin epicatechin and resveratrol with human erythroyte NADH-cytochrome b5 reductase, which is a component of PMRS and together with the identification of minimum pharmacophoric feature using Pharmagist.

**Results:**

The derived common minimum pharmacophoric features show the presence of minimum bioactive component in all the selected polyphenols. Our results confirm wet lab findings which show that these polyphenols have the ability to interact and donate protons to the Human NADH-cytochrome b5 reductase.

**Conclusion:**

With the help of these comparative results of docking simulation and pharmacophoric features, novel potent molecules can be designed with higher efficacy for activation of the PMRS system.

## Background

The property of erythrocytes to reduce membrane impermeant anions was first reported by Orringer and Roer [1]. Later researches established the existence of trans-membranous NADH dehydrogenases in several other cell types [2,3]. Evidence is now clear for the presence of a trans-plasma membrane electron transport or plasma membrane redox system (PMRS) in all organisms including bacteria, yeast, animals and plants [4,5]. It is accepted that PMRS is involved in transferring reducing equivalents from intracellular donors to extracellular acceptors mainly oxidized ascorbate. In this way the PMRS helps the cells to respond to changes in redox potential thereby regulating a variety of physiological functions including cell metabolism, ion channels, growth and death [6,7].

The PMRS has extensively been studied in erythrocytes basically due to the fact that erythrocytes lack mitochondria and PMRS is the only mechanism for trans-plasma membrane electron transport. Importantly, erythrocytes encounter a variety of oxidants in the blood during their life span. Recent reports show that erythrocyte PMRS plays an important role in providing protection against oxidative stress during human aging [8,9] and in type 2 diabetes mellitus [10]. The basic structure of PMRS includes three major entities: the intracellular electron donor species, electron carrier proteins and oxidoreductases and extracellular electron acceptors. An important enzyme of PMRS in erythrocyte is the cytochrome b_5 _reductase (EC 1.6.2.2).

Cytochrome b_5 _reductase is encoded by the CYB5R3 locus located on chromosome 22q 13-qter (287). The tertiary folding structure of human cyt b_5 _red, revealed by X-ray crystallography shows similarity with other flavin-linked oxido reductases such as ferredoxin: NADP+reductase and phthalate dioxygenase reductase [11]. Cyt b_5 _reductase contains two functional lobes: a flavin adenine dinuceotide FAD-binding amino terminal domain (residues 33-147) and NADH-binding carboxyl end domain (residues 148-170). The two domains are linked by a hinge region (residues 148-170), which is critical for the protein conformation and enzymatic activity. Cyt b_5 _red. catalyses one-electron reduction reactions in association with FAD and cytochrome b_5_.

**Figure 1:**
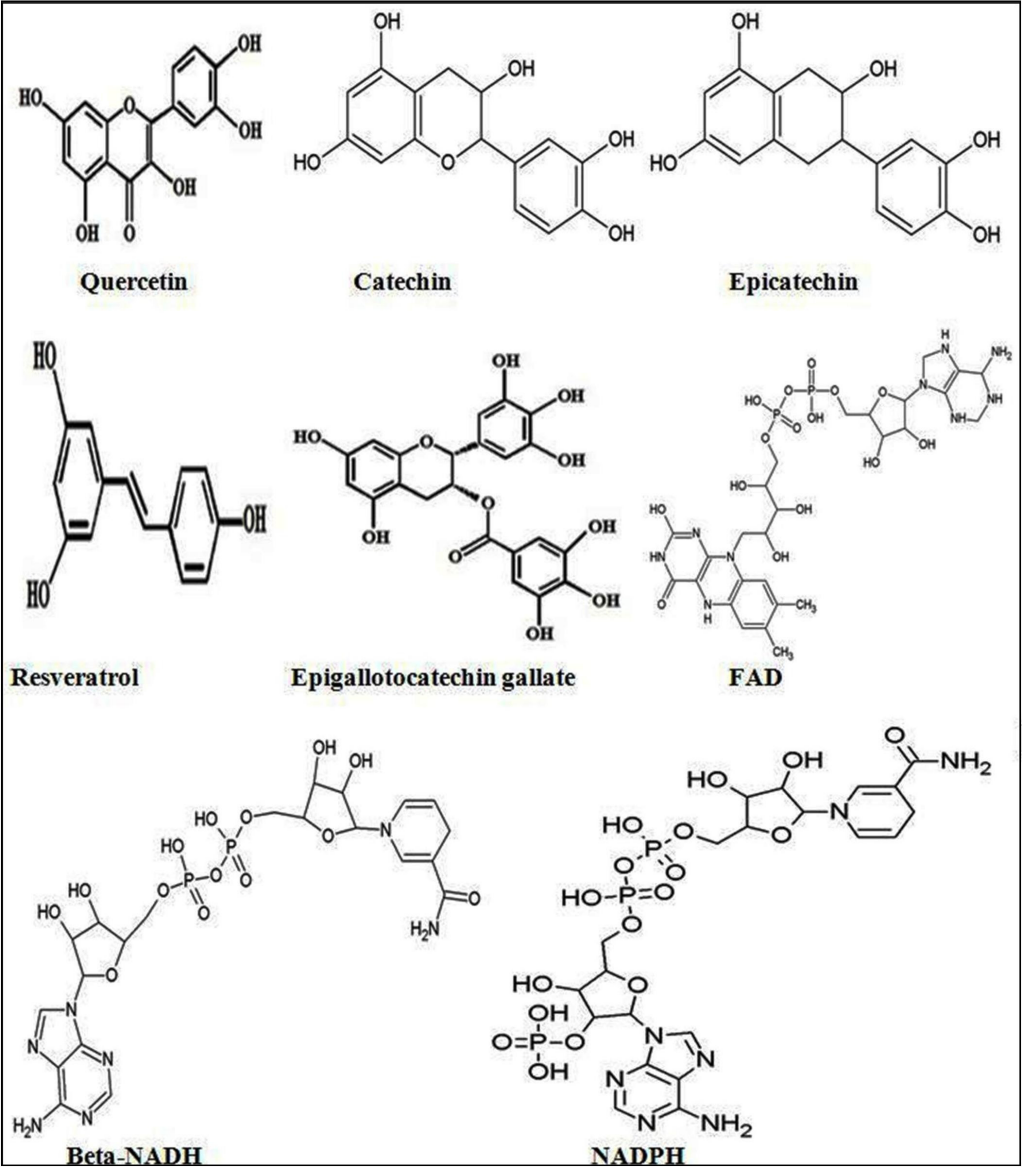
**2-D structure of selected polyphenols (quercetin, catechin, epicatechin, resveratrol and EGCG), FAD, beta-NADH and NADPH**.

In erythrocytes, cytochrome b5 reductase primarily helps in maintaining hemoglobin in its reduced state and also plays a crucial role in reducing extracellular ascorbate-free radical to ascorbate. Earlier it was proposed that only NADH or NADPH provided reducing equivalents to PMRS, however, now it is acknowledged that some polyphenols and ascorbate also have the ability to donate reducing equivalents to PMRS. It is now known that resveratrol, quercetin, myricetin, and epigallocatechin gallate (EGCG) may be taken up by erythrocytes from the plasma and actively promote PMRS activity [12,13]. In view of the important role of PMRS during aging and the emerging opinion that activation of erythrocyte PMRS may be an effective anti-aging strategy [14], this study was undertaken to determine the comparative molecular binding indices of some polyphenols (quercetin, catechin, epicatechin, resveratrol and EGCG together with FAD, NADPH and NADH (Figure 1) with an important component of erythrocyte PMRS, the cytochrome b5 reductase, through computational docking simulation using Molegro Virtual Docker (MVD) [15], Glide module (supplied by Schrödinger suite) [16] and ligand-based pharmacophoric feature derivation using PharmaGist server [17].

## Methods

### Protein structure and preparation

Three-dimensional X-ray crystallized structure of Human NADH-cytochrome b5 reductase (PDB: 1UMK, resolution = 1.75 Å) was downloaded from the Protein Data Bank [18,19]. The downloaded protein has single chain A with 275 residues together with FAD-binding region and contains bound FAD as a ligand molecule. It also contains 753 water molecules of crystallization. The protein structure was prepared using the protein preparation module of Schrödinger software [20]. The co-crystallized ligands and water molecules were removed. Some residues and side chain atom are missing in crystallized structure of protein that was modeled using Prime 2.2.108 followed by refinement of the protein structure. The final modeled protein was taken as receptor protein and found the most suitable-binding site using sitemap script of Schrödinger. On the basis of priority of site, FAD-binding site has been selected for docking with the FAD, NADPH, beta-NADH, catechin, quercetin, epicatechin, EGCG and resveratrol.

### Ligands structure preparation

All the selected ligands were assigned an appropriate bond order using the LigPrep 2.4.107 script and converted to .mae format (Maestro, Schrödinger, Inc.) followed by optimization by means of the OPLS_2005 force field [21].

### Experimental

#### Docking study with MVD

It is an automated docking software with fast processing. The preparation of selected polyphenols and protein were done using default parameters, which automatically adds the missing hydrogen atoms. The software has module to create surface over receptor molecule and to give possible binding site for its activity. The active site region of receptor Human NADH-cytochrome b5 reductase protein was chosen for docking, which is already known from literature with the selected polyphenols. It gives ten conformations for each ligand and returns five outputs with MoleDockScore and other thermodynamically calculated values. The MoleDockScore is an anonymous value on which we have to suggest the best docked ligand with its conformation. It also shows hydrogen bond information together with other thermodynamic values, which suggest the formation of stable complex between ligand and receptor molecule [15].

#### Docking study with glide

The Protein ligand docking studies were performed using Maestro 9.1.107. Default parameters were selected with Glide Extra Precision (XP Glide), version 4.5.19. After the complete preparation of ligands and protein for docking, receptor-grid files were generated. For running the grid generation module we have scaled van der Waal radii of receptor atoms by 1.00 Å with a partial atomic charge of 0.25. A grid box of size 25 × 25 × 25 Å with coordinates X = 37.955433, Y = -6.749032 and Z = 39.920372 was generated at the centroid of the FAD-binding site predicted by sitemap script of Schrödinger suite 10.0. After the formation of receptor-grid file, flexible ligands with rigid receptor docking were performed. Glide generates conformations internally and passes these through a series of filters. The final energy evaluation is done with GlideScore and a single best pose is generated as the output for a particular ligand [16].

#### Pharmacophoric study

It is highly efficient method for the derivation of a minimum pharmacophoric features which is spatial arrangement of physico-chemical properties in a set of ligand, essential for the interaction with a specific receptor. It takes three dimensional structure of set of ligands as an input to multiply align flexible ligands in a deterministic manner and to focus on the input ligands. It searches shared large common substructure for the detection of both outer molecules and alternative binding modes and finally derived pharmacophoric features shared by a large number of ligand molecules as an output [17].

## Results and discussion

The binding site cavity detection and docking simulation was performed by using two different docking softwares, namely MVD and Glide simulation module (supplied by Schrödinger suite) for the selected polyphenols together with FAD NADPH and beta-NADH at Human NADH-cytochrome b5 reductase.

The results obtained using MVD and Glide, shown in terms of MoleDockScore; H-bonding energy and Glide score; LipophilicEvdW enrgy; HBond energy; Electro energy respectively are given in Table 1 and 2.

**Table 1:** Comparative docking simulation result of selected polyphenols, NADPH and beta-NADH with Human NADH-cytochrome b5 reductase together with FAD, ligand from X-ray Crystallized data of protein data bank (1umk.pdb) using MVD

Serial Number	Ligands	MoleDockScore	H-bonding energy
1.	FAD	-232.638	-20.532
2.	NADPH	-209.954	-13.985
3.	beta-NADH	-208.235	-13.506
4.	EGCG	-131.595	-9.012
5.	Quercetin	-113.611	-10.033
6.	Catechin	-110.472	-9.063
7.	Epicatechin	-102.952	-14.638
8.	Resveratrol	-102.074	-10.272

**Table 2:** Comparative docking simulation result of selected polyphenols, FAD, NADPH and beta-NADH with Human NADH-cytochrome b5 reductase using Glide docking simulation software

Serial number	Ligands	GScore	Lipophilic EvdW	HBond	Electro
1	FAD	-12.18	-5.07	-2.14	-0.71
2	NADPH	-10.86	-4.45	-2.13	-0.68
3	beta-NADH	-10.48	-4.43	-1.72	-0.79
4	EGCG	-9.10	-4.13	-1.33	-0.51
5	Catechin	-7.83	-4.41	-1.75	-0.95
6	Quercetin	-7.82	-4.19	-1.58	-0.94
7	Epicatechin	-7.57	-3.12	-1.33	-1.07
8	Resveratrol	-4.29	-3.8	-1.5	-0.54

Where ***GScore*: **It is GlideScore called as Docking Score

***LipophilicEvdW*: **It is term derived from hydrophobic grid potential and fraction of the total protein-ligand Van der Waals energy; ***HBond*: **Hydrogen-bonding term.

**Electro: **This term represents Electrostatic rewards

The comparative result of docking simulation given in Tables 3 and 4, shows active site residues and proves that a number of hydrogen bonds are involved in interaction between selected polyphenols, FAD, NADPH and beta-NADH with the receptor Human NADH-cytochrome b5 reductase.

**Table 3:** Human NADH-cytochrome b5 reductase protein residues interact with selected polyphenols, NADPH and beta-NADH using MVD (highlighted residues are involved in H-bonding interaction with ligands) and FAD from X-ray Crystallized data of protein data bank

**Serial Number**.	ligands	Interacting residues of receptor Human NADH-cytochrome b5 reductase	No. of H-bond interaction
1.	FAD	Arg91, Pro92, Tyr93, Val108, Ile109, Lys110, Tyr112, Phe113, Phe120, Gly123, Gly124, Lys125, Ser127, Thr181, Thr184	13
2.	NADPH	His77, Arg91, Pro92, Tyr93, Thr94, Val108, Ile109, Try112, His117, Phe120, Gly123, Ser127, Thr181, Thr184, Pro185	12
3.	beta-NADH	Arg91, Pro92, Tyr93, Thr94, Val108, Ile109, Lys110, Phe113, His117, Gly123, Gly124, Lys125, Thr181, Thr184	10
4.	EGCG	Arg91, Tyr93, Tyr112, Phe113, His17, Phe120, Gly123, Gly124, Lys125	5
5.	Catechin	His77, Pro92, Tyr93, Thr94, Val108, Ile109, Thr184, Pro185, Phe300	6
6.	Quercetin	His77, Pro92, Tyr93, Thr94, Val108, Ile109, Thr181, Thr184, Cys273, Phe300	7
7.	Epicatechin	Pro92, Tyr93, Thr94, Val108, Ile109, Lys110, Thr181, Thr184, Pro185, Phe300	8
8.	Resveratrol	His77, Pro92, Tyr93, Thr94, Val108, Ile109, Lys110, Thr181, Thr184, Pro185, Phe300	5

Where MoleDock Score is molegro docking scoring function or Energy Score (E_Score_)

**Table 4:** Human NADH-cytochrome b5 reductase protein residues interact with selected polyphenols, NADPH, beta-NADH using Glide docking simulation software (highlighted residues are H-bonding interacting residues) and FAD from X-ray Crystallized data of protein data bank

**S. No**.	ligands	Interacting residues of receptor Human NADH-cytochrome b5 reductase	No. of H-bond interaction
1.	FAD	Arg91, Pro92, Tyr93, Thr94, Val108, Ile109, Lys110, Phe113, Phe120, Gly123, Gly124, Lys125, Met126, Ser127, Gly179, Gly180, Thr181, Thr184	17
2.	NADPH	Thr94, Lys110, Try112, His117, Gly179, Gly180, Thr181, Thr184, Gln210, Cys273, Pro275	8
3.	beta-NADH	Tyr93, Lys110, Tyr112, Gly179, Gly180, Thr181, Gln210, Asp239, Phe251, Val252, Pro275	8
4.	EGCG	His117, Asn209, Gln210, Asp239, Phe251, Met278	4
5.	Catechin	Lys110, Tyr112, Gly180, Ala208, Asn209, Gln210, Phe251, Val252, Pro275	6
6.	Quarcetin	Lys110, Tyr112, Ala208, Asn209, Asp239, Phe251	4
7.	Epicatechin	Lys110, Tyr112, Gly180, Ala208, Gln210, Asp239, Phe251, Val252, Pro275	7
8.	Reserveratrol	Lys110, Tyr112, Ala208, Gln210, Phe251, Val252	3

The binding affinity of selected polyphenols, NADPH, beta-NADH and FAD at the active site of Human NADH-cytochrome b5 reductase using MVD and Glide in decreasing order is: FAD>NADPH>beta-NADH>EGCG>quercetin>catechin>epicatechin>resveratrol and FAD>NADPH>beta-NADH>FAD>NADPH>beta-NADH>EGCG>catechin>quercetin>epicatechin>resveratrol respectively.

**Figure 2:**
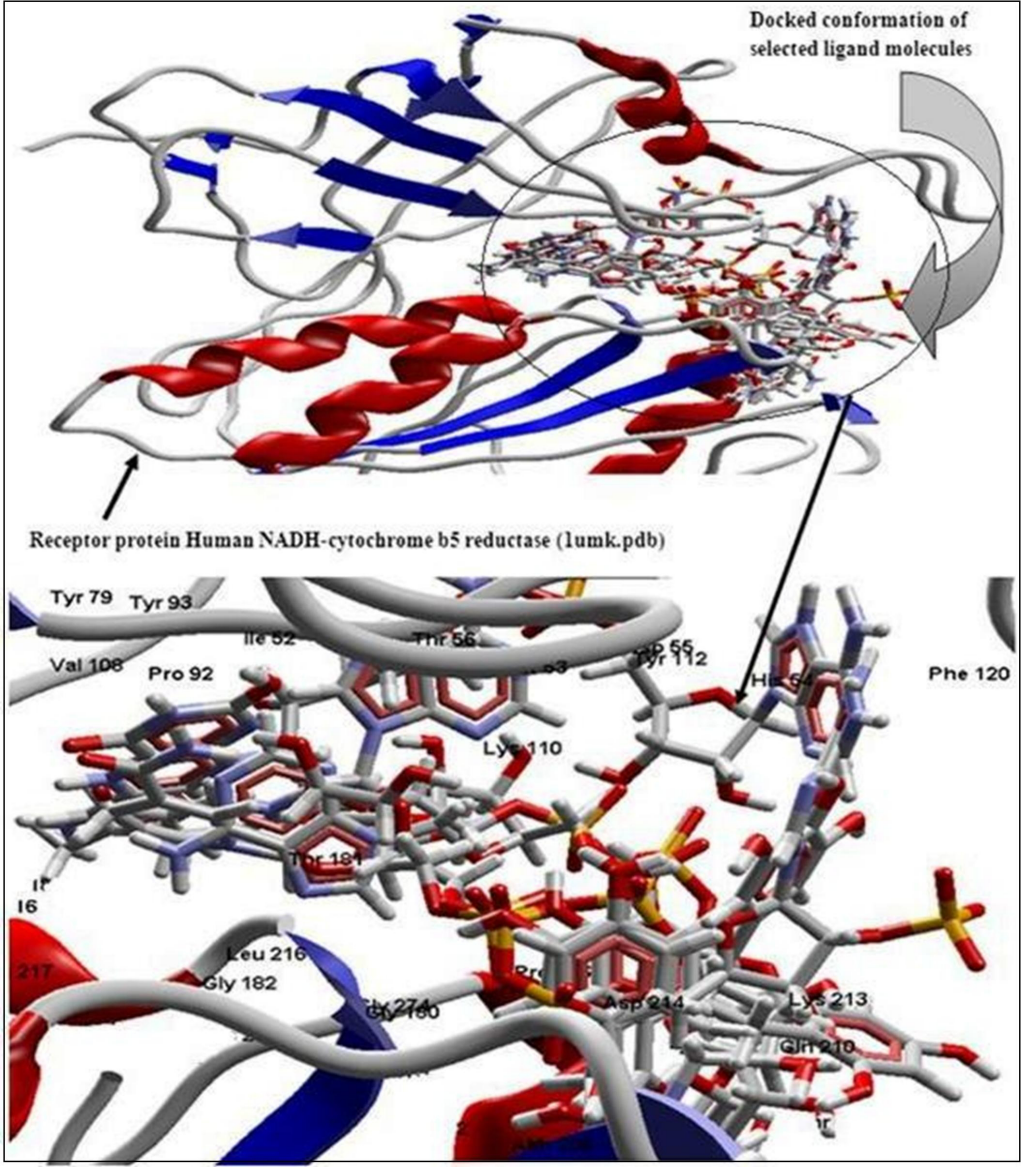
**Secondary structure (cartoon) representation of the active site of receptor human NADH-cytochrome b5 reducatse protein with docked conformation of selected ligand molecules NADPH, beta-NADH, EGCG, quercetin, catechin, epicatechin, resveratrol together with FAD (ligand from crystal structure of 1umk.pdb) using Glide**.

**Figure 3:**
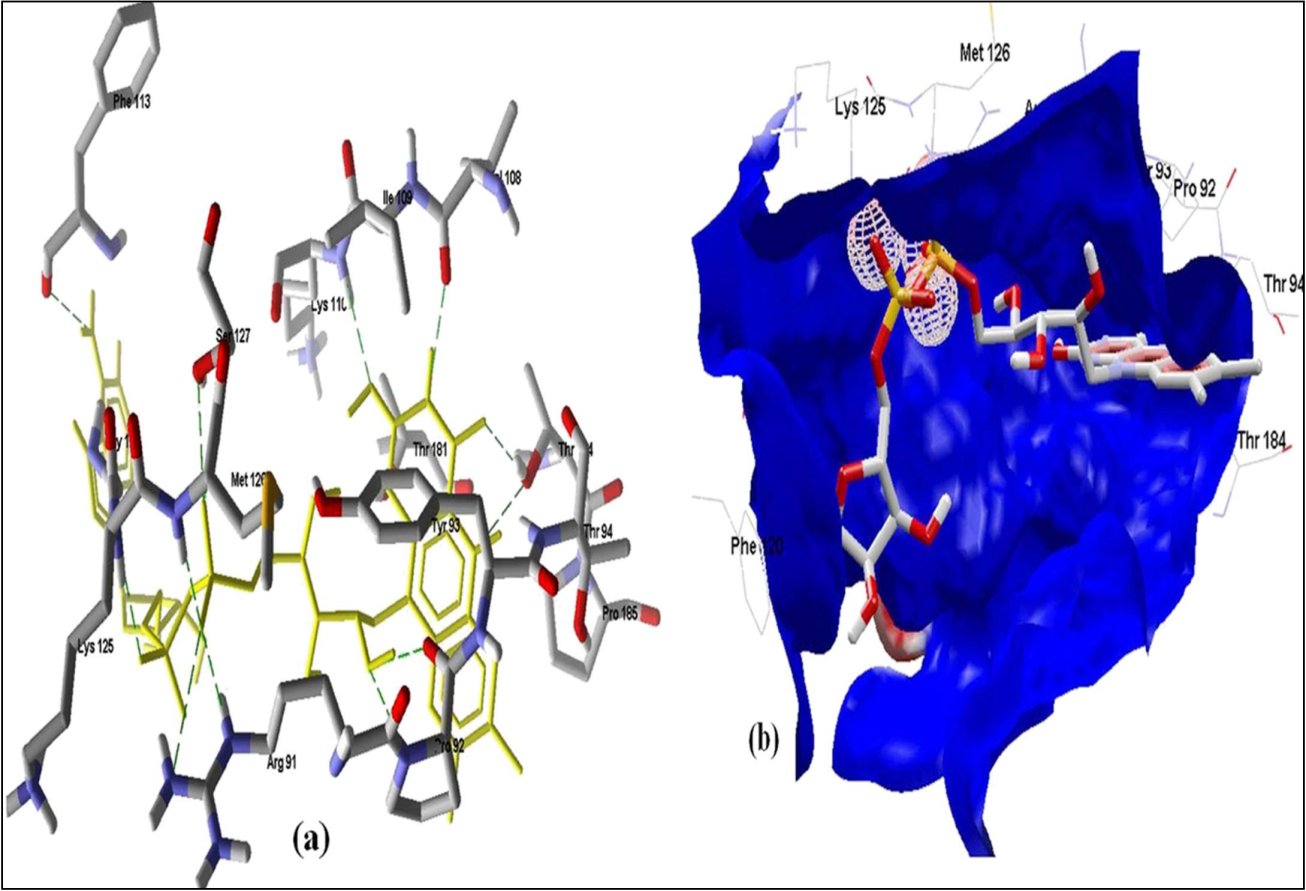
**Docked conformation of hydrogen bonding view and 3.b with Electrostatic interaction of FAD with interacting amino acids of human NADH-cytochrome b5 reducatse protein at the active site cavity**.

**Figure 4:**
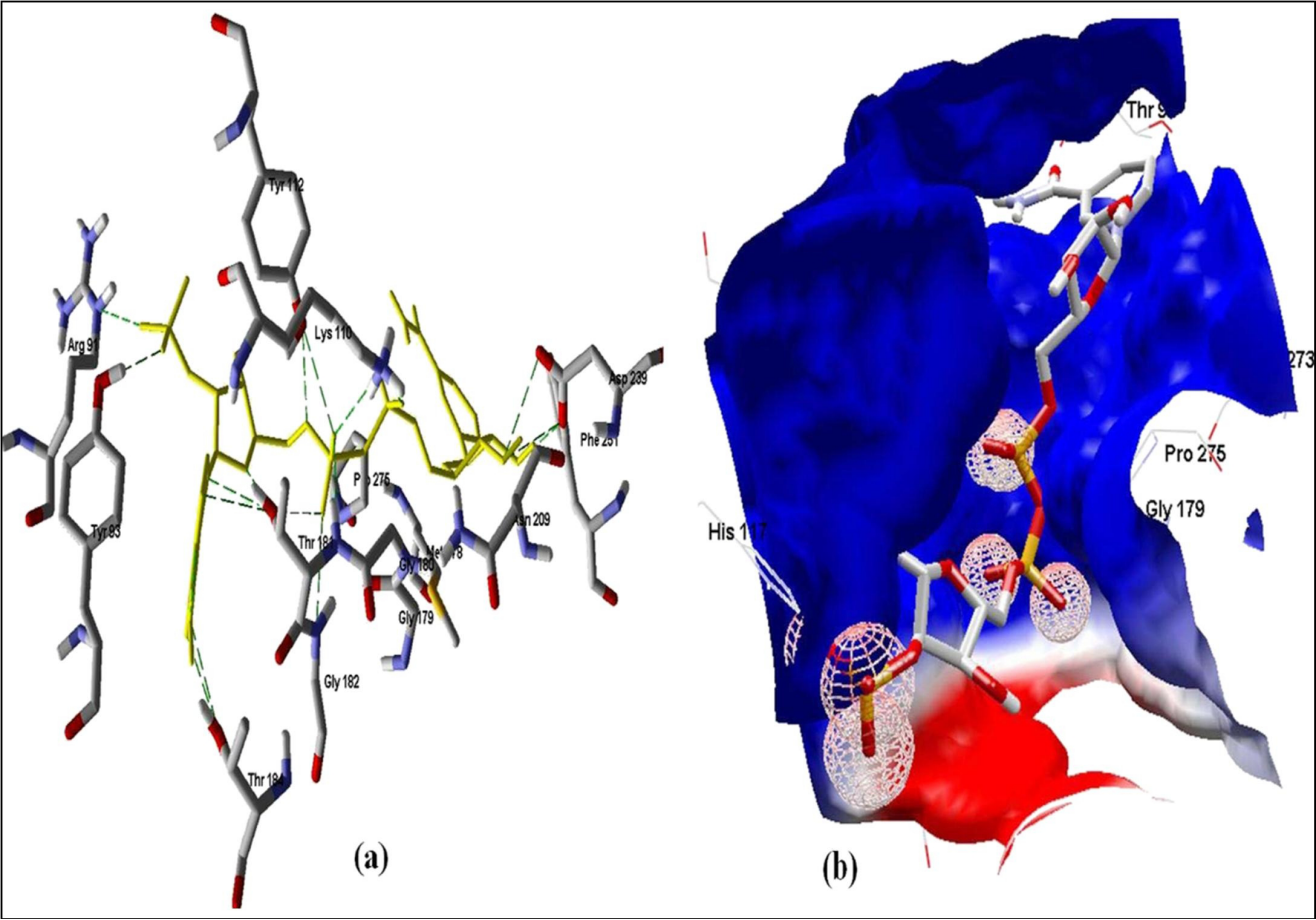
**Docked conformation of hydrogen bonding view and 4.b with Electrostatic interaction of NADPH with interacting amino acids of human NADH-cytochrome b5 reducatse protein at the active site cavity**.

**Figure 5:**
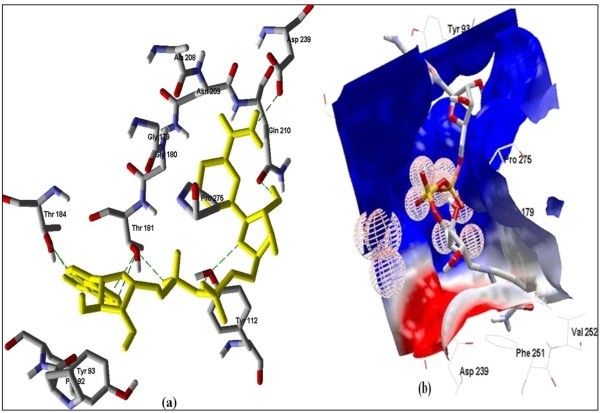
**Docked conformation of hydrogen bonding view and 5.b with Electrostatic interaction of beta-NADH with interacting amino acids of human NADH-cytochrome b5 reducatse protein at the active site cavity**.

**Figure 6:**
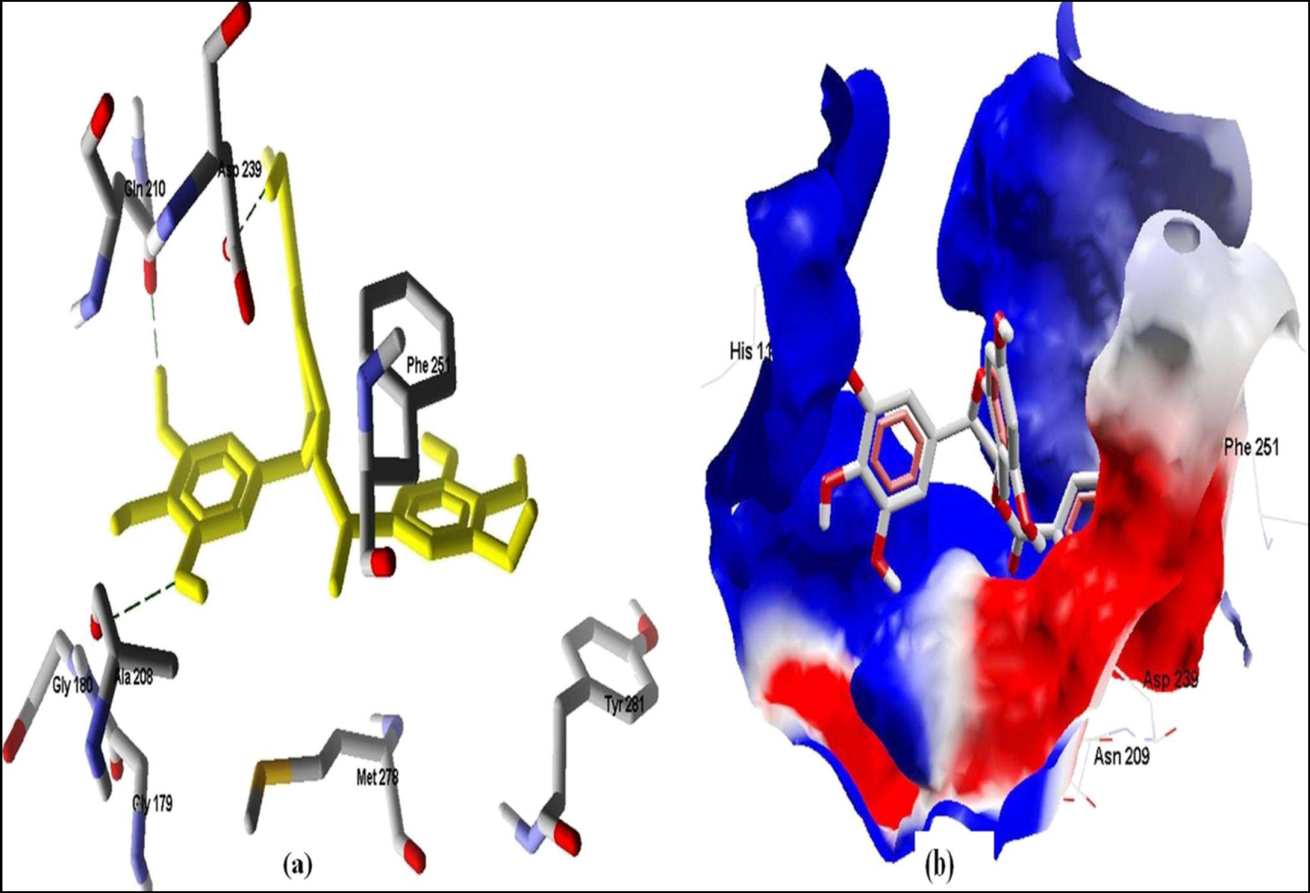
**Docked conformation of hydrogen bonding view and 6.b, Electrostatic interaction of EGCG with interacting amino acids of human NADH-cytochrome b5 reducatse protein at the active site cavity**.

**Figure 7:**
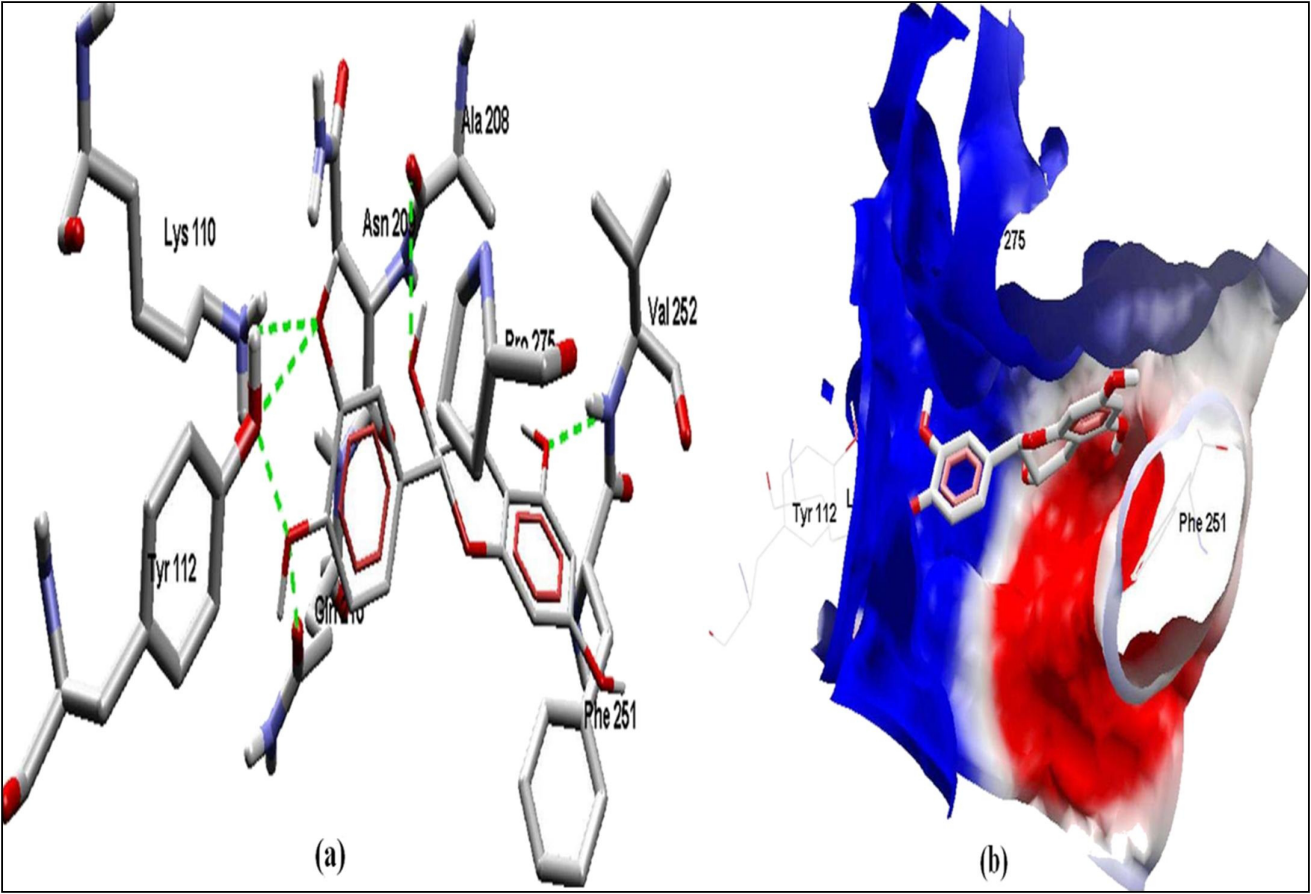
**Docked conformation of hydrogen bonding view and 7.b with Electrostatic interaction of Catechin with interacting amino acids of human NADH-cytochrome b5 reducatse protein at the active site cavity**.

**Figure 8:**
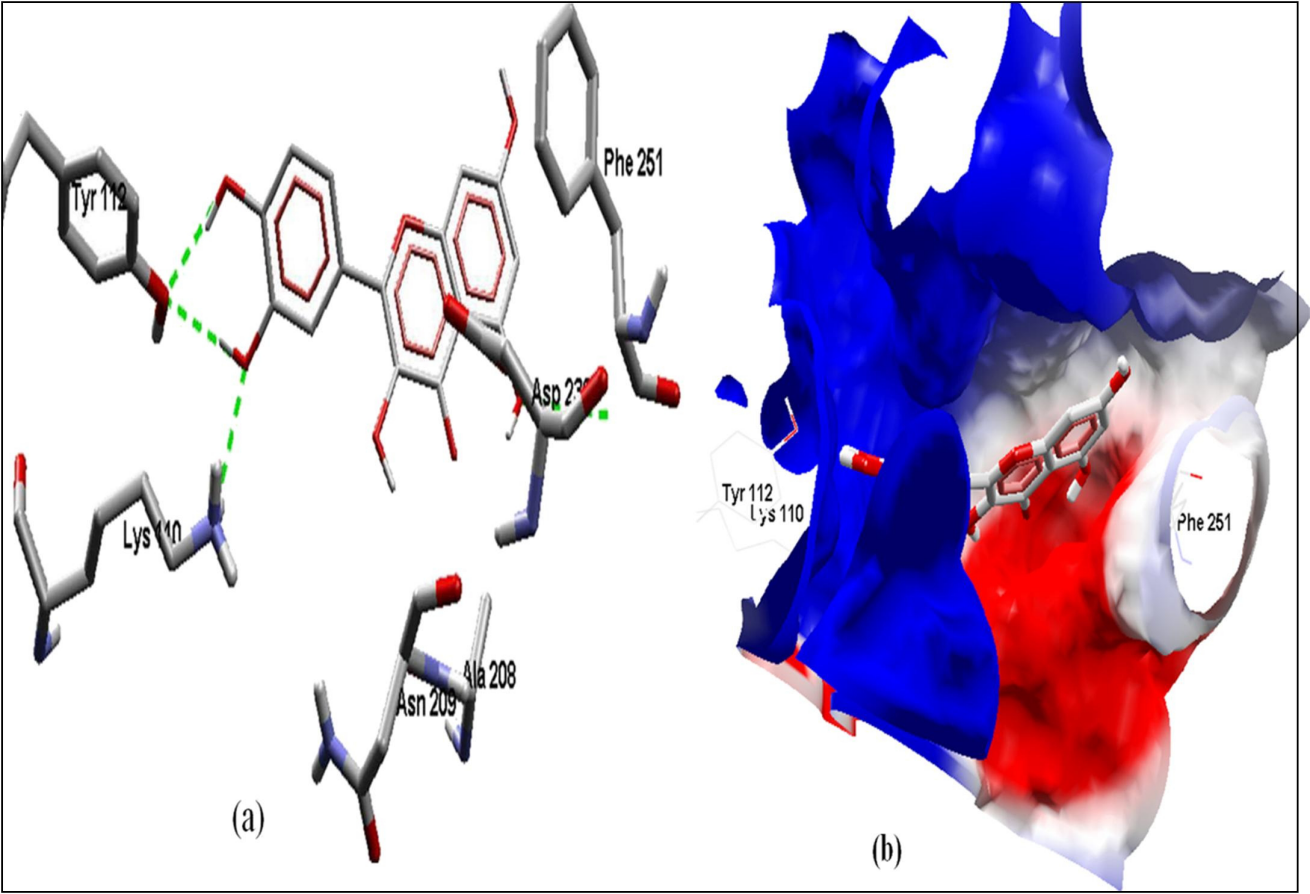
**Docked conformation of hydrogen bonding view and 8.b with Electrostatic interaction of Quercetin with interacting amino acids of human NADH-cytochrome b5 reducatse protein at the active site cavity**.

**Figure 9:**
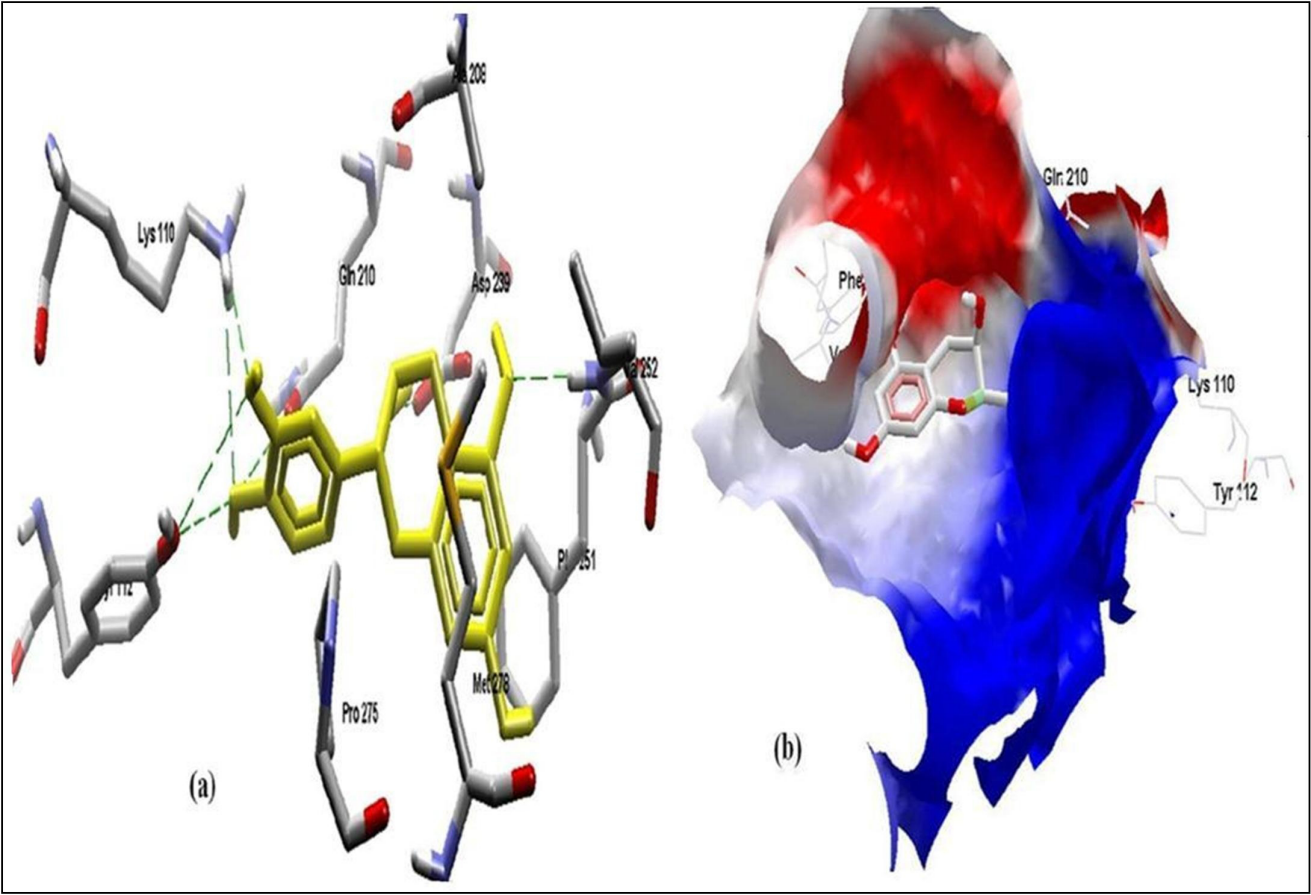
**Docked conformation of hydrogen bonding view and 9.b with Electrostatic interaction of Epicatechin with interacting amino acids of human NADH-cytochrome b5 reducatse protein at the active site cavity**.

**Figure 10:**
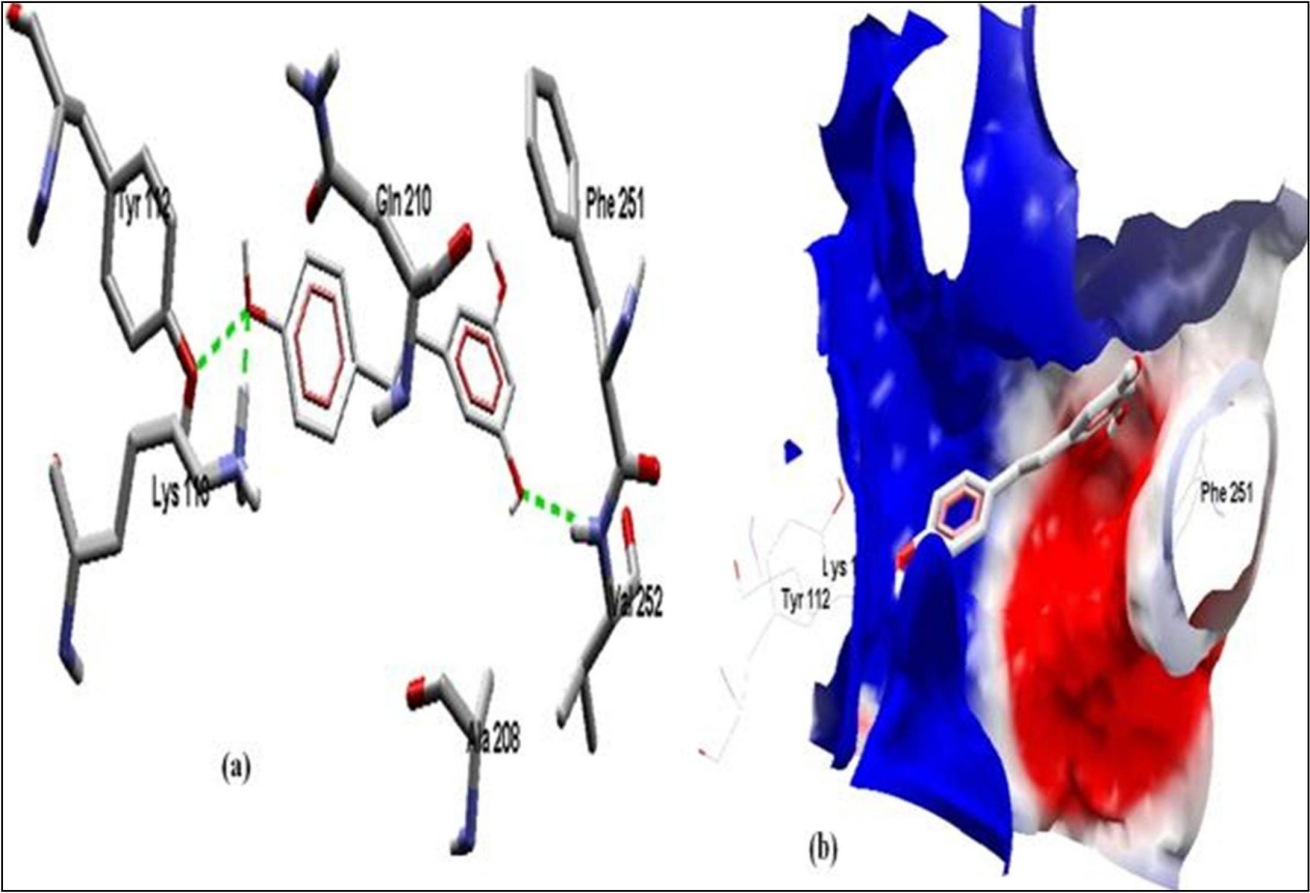
**Docked conformation of hydrogen bonding view and 10.b with Electrostatic interaction of Resveratrol with interacting amino acids of human NADH-cytochrome b5 reducatse protein at the active site cavity**.

The Figure 2 of Glide docking simulation results shows low energy bound conformation of selected polyphenols, NADPH, beta-NADH and together with FAD (ligand from crystal structure of PDB:1UMK) at the active site of Human NADH-cytochrome b5 reductase. The low energy bound conformation of selected ligands shows hydrogen bonding and electrostatic interactions as shown in Figure 3 (a, b), 4 (a, b), 5 (a, b), 6(a, b), 7 (a, b), 8(a, b), 9 (a, b) and 10(a, b) for FAD, NADPH, beta-NADH, epigallocatechin gallate, catechin, quarcetin, epicatechin and resveratrol respectively.

Figure 2 of Glide docking simulation results shows low energy bound conformation of selected polyphenols, NADPH, beta-NADH, and together with FAD (ligand from crystal structure of PDB:1UMK) at the active site of Human NADH-cytochrome b_5 _reductase. The low energy bound conformation of selected ligands shows hydrogen bonding and electrostatic interactions as shown in Figures 3a, b, 4a, b, 5a, b, 6a, b, 7a, b, 8a, b, 9a, b, and 10a, b for FAD, NADPH, beta-NADH, EGCG, catechin, quarcetin, epicatechin, and resveratrol, respectively

**Figure 11:**
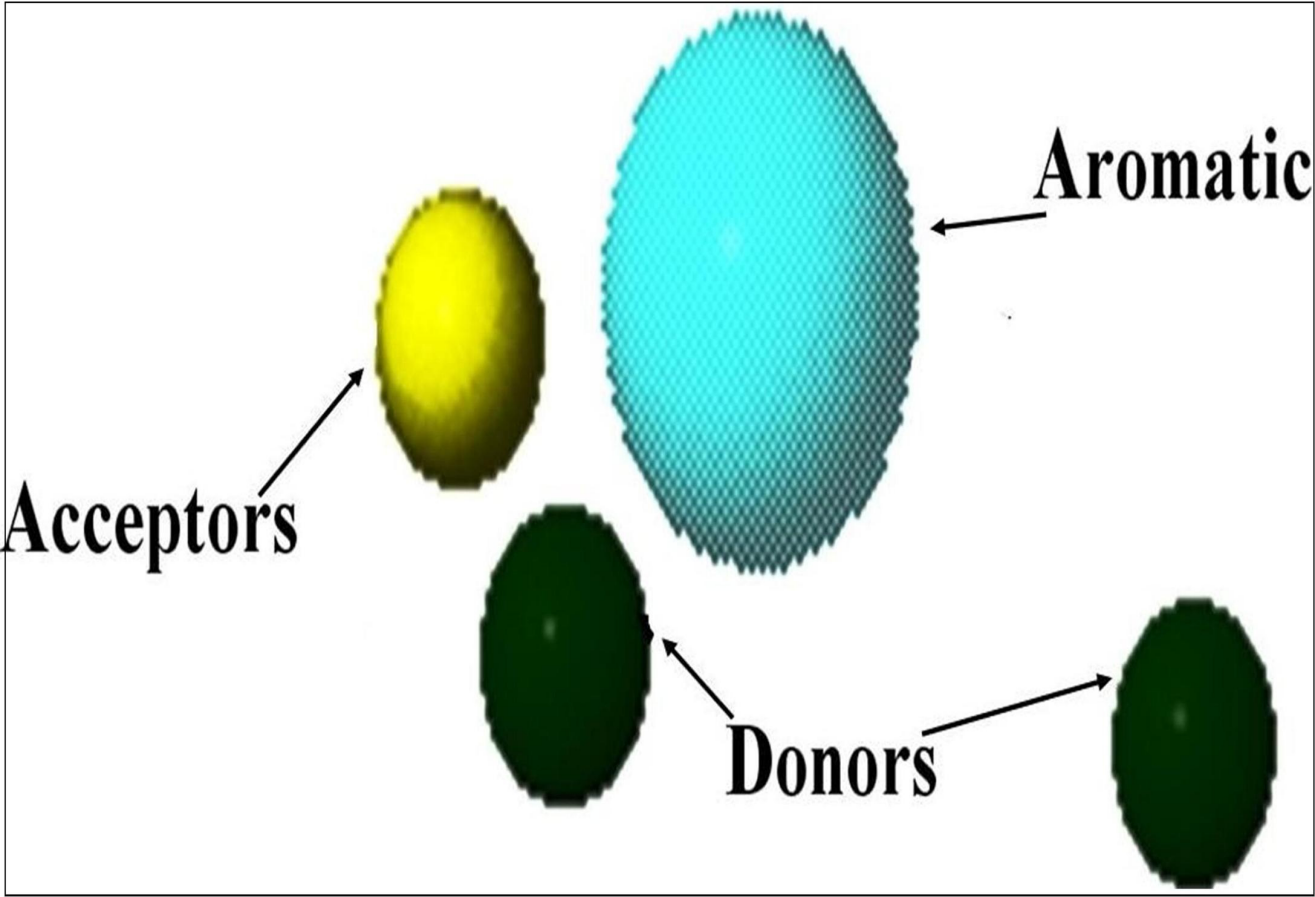
**Derived Common pharmacophoric features for all the selected polyphenols (NADPH, beta-NADH, EGCG, Quercetin, Catechin, Epicatechin, Resveratrol and FAD) using PharmaGist sever**.

Computational methods provide aids for not only designing and interpretation of hypothesis-driven experiments in the field of drug discovery research but may also be used to compare in vitro results for rapid generation of new hypotheses. The binding affinity was higher for FAD because it is a natural ligand of the receptor protein having highest number of hydrogen bonds. The formation of hydrogen bonds provides additional force to stabilize the ligand-protein complex required for the activity of the Human NADH-cytochrome b5 reductase. The results obtained using two different docking simulation softwares were compared and their findings were common, which also strongly supports our *in silico *findings. An analysis of pharmacophoric features of the docked conformation for all the selected polyphenols, NADPH, beta-NADH and FAD, in the study provided the minimum common phormacophoric features shown in Figure 11, which include one aromatic ring, two donor atoms and one acceptor atom derived from PhrmaGist server [17]. The presence of minimum common pharmacophoric features in all the selected polyphenols proves the wet lab findings [12,13] which show that these polyphenols have the ability to interact and donate protons to the Human NADH-cytochrome b5 reductase.

## Conclusion

Given the importance of human PMRS system during human aging and life span determination [8,9,22], our findings provide important insight into the docking and binding characteristics of the selected polyphenols on the Human NADH-cytochrome b5 reductase. With the help of these comparative results for docking simulation and pharmacophoric features, molecules having higher electron donor/acceptor efficacy for activation of the PMRS system can be designed. It is significant that activation of PMRS is being viewed as a putative mechanism for designing anti-aging agents [14]. Our in silico study may also lead to important information regarding the use of polyphenols as immunomodulating agents.

## Abbreviations

EGCG: Epigallocatechin gallate; PMRS: Plasma membrane redox system.

## Competing interests

The authors declare that they have no competing interests.

## Authors' contributions

RKK carried out docking simulation study, DVS performed pharmacophoric study, KM supervised docking and pharmacophoric studies, and SIR conceived the study, drafted the manuscript. All authors read and approved the final manuscript.
